# Older adults' attitudes about continuing cancer screening later in life: a pilot study interviewing residents of two continuing care communities

**DOI:** 10.1186/1471-2318-6-10

**Published:** 2006-08-03

**Authors:** Carmen L Lewis, Christine E Kistler, Halle R Amick, Lea C Watson, Debra L Bynum, Louise C Walter, Michael P Pignone

**Affiliations:** 1 Division of General Medicine and Clinical Epidemiology, The University of North Carolina at Chapel Hill Chapel Hill, NC, USA; 2 Department of Family Medicine, University of Michigan, USA; 3 Robert Wood Johnson Clinical Scholars Program, The University of North Carolina at Chapel Hill, USA; 4 Department of Psychiatry, The University of North Carolina at Chapel Hill, USA; 5 Division of Geriatrics, The University of North Carolina at Chapel Hill, USA; 6 Division of Geriatrics, San Francisco VA Medical Center, University of California, San Francisco, USA

## Abstract

**Background:**

Individualized decision making has been recommended for cancer screening decisions in older adults. Because older adults' preferences are central to individualized decisions, we assessed older adults' perspectives about continuing cancer screening later in life.

**Methods:**

Face to face interviews with 116 residents age 70 or over from two long-term care retirement communities. Interview content included questions about whether participants had discussed cancer screening with their physicians since turning age 70, their attitudes about information important for individualized decisions, and their attitudes about continuing cancer screening later in life.

**Results:**

Forty-nine percent of participants reported that they had an opportunity to discuss cancer screening with their physician since turning age 70; 89% would have preferred to have had these discussions. Sixty-two percent believed their own life expectancy was not important for decision making, and 48% preferred not to discuss life expectancy. Attitudes about continuing cancer screening were favorable. Most participants reported that they would continue screening throughout their lives and 43% would consider getting screened even if their doctors recommended against it. Only 13% thought that they would not live long enough to benefit from cancer screening tests. Factors important to consider stopping include: age, deteriorating or poor health, concerns about the effectiveness of the tests, and doctors recommendations.

**Conclusion:**

This select group of older adults held positive attitudes about continuing cancer screening later in life, and many may have had unrealistic expectations. Individualized decision making could help clarify how life expectancy affects the potential survival benefits of cancer screening. Future research is needed to determine whether educating older adults about the importance of longevity in screening decisions would be acceptable, affect older adults' attitudes about screening, or change their screening behavior.

## Background

Although cancer is an important cause of morbidity and mortality in older adults [1], the incidence of other serious conditions also increases with age. These competing causes of mortality decrease the likelihood that an older individual could experience a survival benefit from cancer screening [2] because cancers are relatively slow growing. Therefore, those at advanced age and with multiple co-morbid conditions could undergo screening and not live long enough to realize a mortality benefit [3]. Available evidence from cancer detection trials in middle aged patients suggests that at least 5 years of life expectancy is needed to reduce disease specific mortality for breast and colon cancer [4,5], and for prostate cancer it is estimated that men should have a life expectancy of 10 or more years [6,7].

Older adults' life expectancy varies widely depending upon age and health states [3]. To accommodate the wide variation in life expectancy, individualized decision making is recommended for cancer screening decisions later in life [3,8,9]. During this process, older adults and their physicians consider the risk of dying from cancer, dying from other causes besides cancer, the risks and benefits of screening, and personal preferences [3].

For individualized decision making to be implemented in clinical practice, older adults need an opportunity to obtain and understand information necessary for informed decision about whether to continue or stop cancer screening [10,11]. This information involves concepts that may be difficult to understand, such as, competing causes of mortality and delayed benefit from screening. A nurse led program to promote individualized decision making in a continuing care retirement community demonstrated a decrease in screening behaviors over one year; although these results were not statistically significant the number of participants was small [12]. These results suggest that education could promote individualized decision making, but it did not examine attitudes toward the information thought necessary for an informed decision or its relevance to decision making. If older adults do not consider this information relevant to their decision making or would not consider stopping cancer screening, these attitudes could serve as an important barrier to the acceptance of individualized decision making.

Therefore, we undertook this study to better understand the perspectives of older adults in regards to individualized decision making about cancer. Our intent was to assess aspects of cancer screening decisions unique to older adults, including considerations about the possibility of stopping screening. We interviewed older adults to determine 1) if older adults reported the opportunity to engage in individualized decision making with their physicians 2) their attitudes about information important for individualized decision making, and 3) their attitudes about continuing cancer screening later in life.

## Methods

### Setting and participants

We recruited volunteers from the independent living section of two local continuing care retirement communities affiliated with the University of North Carolina Program on Aging. We used this setting because these residents have few structural or financial barriers to health care and we believed that most would have been screened regularly for cancer, have ongoing relationships with primary care doctors, and have had discussions about cancer screening with their doctor.

Residents were recruited through flyers asking for volunteers to participate in a study about cancer screening in older adults. Inclusion criteria were age greater than 70, English speaking, and residency in the independent living facilities of these communities. Exclusion criteria were cognitive impairment determined by a score of greater than or equal to 2 on Callahan's six item screen [13].

One hundred twenty four residents called to inquire about the study from approximately 720 residents in both facilities. One hundred twenty were interviewed, but 1 interview was incomplete, 3 interviewees were later excluded (2 age < 70 and 1 lived in assisted living area). Written informed consent was obtained at the time of the interview and the University of North Carolina School of Medicine Committee on Protection of the Rights of Human Subjects approved the study. The study was also approved by the internal review boards of each continuing care retirement community.

### Interviews

One of the investigators (CK) interviewed participants at the continuing care facilities, usually in participants' homes. Interviews were structured with both closed and open-ended questions, and averaged 80 minutes in duration.

To determine participants' recent screening behavior, we asked all participants when they had last been screened for colon cancer; women when was the last time they were screened for breast cancer; and men when was the last time they were screened for prostate cancer. The interviewer asked participants whether they were familiar with screening tests, and if not, provided standardized explanations.

To determine participants' opportunities for individualized decision making with their physicians, we asked participants whether they had discussed cancer screening tests with their physicians since turning age 70, followed by several questions about the information participants may have received. We also asked if their physicians had inquired since they turned age 70 if they wanted to keep getting tests to check for breast/prostate or colon cancer.

Because this is a new area of inquiry, there are no validated questionnaires directly applicable to older adults' attitudes about cancer screening; therefore, we developed our own questions. Questions were based on the informed decision making criteria proposed by Braddock et. al. [10,11]. The content of the attitudinal statements was directed at issues thought to be important for individualized decision making and unique to older adults' decisions. The content of questions about individualized decision making included questions about life expectancy, the importance of physician recommendations, and the accuracy of screening tests (Table 2). We then asked attitudinal questions about continuing cancer screening, first in regard, to the participant and then about others. These included questions about competing causes of mortality, discomfort and hassle of the tests, delayed benefit from screening, and functional and cognitive decline (Table 3). The format of the questions was Likert scale (strongly agree, agree, disagree, strongly disagree). Finally, we asked the following open-ended question about the possibility of stopping cancer screening, *"What might make you stop getting screened for cancer?"*

**Table 1:** Participant Characteristics (n = 116)

	n (%)
Age (mean 81.6 SD 5.2)	Range (71–96)
70–79	42 (36)
80–84	38 (33)
85 or greater	36 (31)
Race	
White	116 (100)
Sex	
Women	78 (67)
Men	38 (33)
Marital Status	
Married or living with partner	62 (53)
Never married	2 (2)
Widowed	48 (41)
Divorced	4 (4)
Education	
12 grade	2 (2)
Some college	18 (15)
College graduate or higher	96 (83)
IADLS	
Independent	110 (95)
Dependent in 1 or more	6 (5)
Health Status	
Excellent to very good	65 (56)
Good	41 (35)
Fair	10 (9)
No. of Co-morbidities	
0	35 (30)
1	35 (30)
2 through 4	46 (40)
Screened for cancer within the last year	
Colon	77 (66)
Breast	66 (85)
Prostate	30 (80)
Ever had false positive cancer screening test	54 (46)
Primary physician is generalist	108 (93)
Seeing primary care doctor for	
More than 5 years	40 (34)
Has had cancer (excluding skin)	45 (39)

**Table 2:** Attitudes about Information Important for Individualized Decision Making

	**Agree n (%)**		
	All	Age >85	Cancer
	n = 116	n = 36	n = 45

I want my doctor to talk with me about how tests for cancer can give the wrong result	108 (94)	32 (89)	43 (95)
I want my doctor to talk with me about whether I want to stop getting tests to check for cancer.	97 (84)	30 (86)	35 (78)
My doctor's estimate of how long I might live is not important in making decisions about cancer screening.	72 (62)	26 (72)	26 (58)
To help make cancer screening decisions, I want my doctor to talk with me about how long he/she thinks I might live.	60 (52)	17 (46)	23 (52)
I think that doctors know for sure if cancer screening helps people over 70.	56 (49)	18 (51)	23 (51)

**Table 3:** Attitudes about Continuing Cancer Screening Later in Life

	**Agree %**		
	All	Age >85	Cancer
	n = 116	n = 36	n = 45

**For themselves**			
I will likely die of some other disease besides cancer.	86 (81)	29 (80)	33 (73)
I will continue cancer screening no matter how uncomfortable the tests are.	84 (77)	28 (78)	35 (77)
I plan to get screened for colon cancer for as long as I live.	76 (72)§	22 (61)	33 (74)
I plan to get screened for breast/prostate cancer for as long as I live.	85 (83)¶	29 (80)	40 (88)
I will consider getting screened for cancer even if my doctor recommends against it.	47 (43)	19 (53)	26 (58)*
It takes several years for cancer screening to benefit people.	28 (25)	10 (29)	13 (29)
I will not live long enough to benefit from cancer screening tests.	15 (13)	10 (28)*	5 (11)
I will not get cancer screening even if my doctor recommends it.	4 (4)	1 (3)	1(2)
Cancer screening is not worth the trouble.	3 (3)	1 (3)	1 (2)
Everyone should get screened for colon cancer for as long as they live.	64 (55)	22 (61)	31 (69)*
Everyone should get screened for breast/prostate cancer for as long as they live.	73 (63)	23 (64)	41 (91)*
Screening for cancer in people over the age of 70 may waste healthcare time and money.	34 (30)	12 (34)	10 (22)
As people get older, other health issues are more important than cancer screening.	56 (50)	18 (50)	16 (36)*
People in nursing homes should not get cancers screening.	30 (26)	9 (25)	9 (21)
People over 70 who are totally dependent on someone else for daily functions such as eating, bathing, and toileting should not get cancer screening.	50 (44)	15 (43)	18 (40)
People with Alzheimer's disease or dementia should not get cancer screening.	51 (44)	18 (50)	18 (41)

*p < 0.05

§n = 105

¶n = 102

To assure that participants understood the content of the questions in they manner they were intended, all questions were pre-tested. We used the "think out loud" cognitive testing technique [14] with 49 participants over 65 years of age at the University of North Carolina Ambulatory Care Clinic. The questions were revised for clarity and meaning in an iterative process during pre-testing.

Standard measures were used to obtain participant demographics. We also asked them to identify their main doctor, how long they had been seeing him/her, their main doctor's specialty and gender, and the number of doctors they had seen in the last year. Current health status and global function were assessed using Katz's modification of the Charlson co-morbidity index [15], and the Instrumental Activities of Daily Living [16].

### Data analysis

STATA 8.0 (College Station, TX.) was used for the quantitative analysis. We used frequencies to describe participant characteristics and responses to the closed-ended questions and statements. Bi-variate comparisons were made using the Fisher's exact tests, after collapsing the four point Likert scales to "agree" and "disagree". For the open-ended questions, three investigators (CK, CL, and HA) developed a coding scheme and independently coded the responses to the open-ended question. Coding was then reviewed by the group and disagreements were resolved by consensus.

## Results

The 116 participants from the two continuing care retirement communities were well educated, most (83%) had college degrees; 76% were women, and 64% were age 80 and over. All were self-sufficient in basic activities of daily living. The majority reported good to excellent health, although 40% reported having 2 to 4 of the health conditions from Katz's modification of the Charlson co-morbidity measure for interviews [15] (history of cardiovascular disease, congestive heart failure, diabetes, pneumonia, chronic obstructive lung disease or asthma, ulcer disease, renal disease, connective tissue disease, cirrhosis or liver damage, and cancer).

Thirty-nine percent of participants reported having had a previous, not current, diagnosis of cancer other than skin cancer. For those who had been diagnosed with cancer, 6 had colon cancer, 18 had breast cancer, and 13 had prostate cancer. Among those who had not been diagnosed with colon, breast, or prostate cancer, 98% reported having been screened for breast or prostate cancer in the last 5 years, 67% within the past year. Similarly, 96% were screened for colon cancer, 67% within the last year. Participants' age 85 and older had similar screening rates to those under age 85; 91% had been screened for colon cancer within the last 5 years, 52% within the last year and 97% had been screened for breast or prostate cancer, 83% within the last year.

### Opportunities for individualized decision making for cancer screening

Forty-nine percent of participants reported that they had discussed one or more cancer screening tests with their physicians since they had turned 70 years of age; of these, most had discussed more than one screening test. Thirty-one percent of all participants reported discussing either breast and colon cancer screening or prostate and colon cancer screening. When asked about the content, 26% of all participants reported discussing how screening tests can sometimes give the wrong result (half of these were discussions about prostate cancer screening), and 1 out of 10 had been told that cancer screening may not benefit some adults. Thirteen percent overall, and 11% of those age 85 and older said they had been asked by their physicians if they wanted to keep getting tested for cancer.

### Attitudes about individualized decision making

Although most participants had not discussed the possibility of stopping cancer screening with their physicians, when prompted 84% said they wanted to have these discussions (Table 2). Despite this desire, almost half (48%) did not want to discuss their life expectancy, and 62% did not think their doctor's life expectancy estimate was important in making cancer screening decisions.

We compared the attitudes of participants' age 85 and older with those younger but found no differences among these groups, although the numbers were small. Similarly, the attitudes of those who had previously been diagnosed with cancer did not differ from those who had not had cancer for these questions.

### Attitudes about continuing cancer screening later in life

Participants' responses to close-ended questions revealed that their attitudes about continued cancer screening were favorable (Table 3). When considering themselves, most participants reported that they would continue screening throughout their lives; 43% would consider getting screened even if their doctors recommended against it. Only 13% thought that they would not live long enough to benefit from cancer screening tests. Eighty-one percent of participants believed that they would die of some other disease besides cancer, and 3 out of 4 believed that the benefit from cancer screening occurred immediately.

When considering others, most believed that those living in nursing homes (74%), those with Alzheimer's disease (66%), or those totally dependent on others (66%) should continue to get screened. Fifty-five percent believed that everyone should get colon cancer screening and 63% believed that everyone should get breast/prostate screening for as long as they live.

In subgroup analyses, the attitudes of participants aged 85 and over were similar to younger participants, as were those with a history of cancer compared to those without such a history. The exceptions were that participants 85 and over were more likely to believe that they would not live long enough to benefit from screening. And, those with cancer were more likely to consider screening even if their doctor recommended against it and were less likely to think that other health issues were more important than cancer screening.

In response to open-ended questions about whether participants had considered stopping screening, 23 participants reported that they had stopped cancer screening, but when asked if they had decided to stop screening entirely only 11 said they had made this decision. The others had stopped one test but continued other screening tests or interrupted their screening schedule but wanted to resume.

### Responses to "What might make you stop cancer screening?"

To better understand the circumstances under which older adults would stop cancer screening, we asked "What might make you stop getting screened for cancer?"

Participants identified several conditions for which cancer screening may no longer be desirable. First, age was an important factor.

"I am ninety-two and I don't intend to prolong this if I don't have to"

"If I got to be really old, I think I would say to heck with it. Like in my nineties."

Deteriorating health, poor quality of life, or nearing death were also thought to be reasons to stop.

"If I were going to die anyhow, from my heart etc, I would want to stop cancer screening"

"If I were doing poorly in every other way, I might say why bother"

"I guess if I thought I were on death's door"

Concerns about screening tests were also cited as reasons to stop:

"If screening methods were proven unreliable or if screening dangers outweigh the possible benefits"

"If I felt the test was unreliable or if early detection did not have much of an effect."

Doctors' recommendations were also cited as important to participants decisions to continue or to stop screening:

"*As far as I know I should continue, so unless my doctor says to stop I will continue, despite the pain."*

"The doctors don't think the colonoscopy is that necessary at my age, and I do what the doctor says"

## Discussion

About half of this select group of older adults reported having had an opportunity for individualized decision making with their physicians regarding cancer screening since turning age 70; however, a majority of participants reported that they would like to have these types of discussions with their physicians. Among those who had discussions, only 13% of all participants said they had been asked if they wanted to continue screening. Although many wanted to discuss the possibility of stopping screening with their physicians, about half preferred not to discuss life expectancy and 62% did not think that physicians' estimates of life expectancy were important in making screening decisions. Their positive responses to attitudinal questions suggest that they strongly believe that continued cancer screening is of benefit to them and to others; only a small number had stopped cancer screening completely; most planned to continue screening throughout their life, and only a minority believed that older adults with severe co-morbidities and resultant functional limitations should stop cancer screening. In most cases, those older than 85 with presumably shorter life expectancies, held similar beliefs to those who were younger. Factors important to participants when deciding about stopping include: age, deteriorating or poor health, concerns about the effectiveness of the tests, and doctors' recommendations.

Many studies have examined cancer screening behavior in adults 64 and over [17-23]. Some studies focused on under-utilization of cancer screening, identifying barriers to screening in older adults in order to promote screening [17,22,24]. Recently, the focus has shifted from a health promotion strategy for all older adults to a strategy of individualized decision making for adults age 75 and over [3,12,25,26]. This strategy takes into account the wide variation in health status for this population and acknowledges the lack of direct evidence supporting screening.

Few studies have examined individualized decision making strategies. Some evidence suggest that education about the pros and cons of screening as an older adult could influence screening behavior [12]. Our findings suggest that there may be some important patient barriers to individualized decision making in clinical practice. Although we chose this well educated population as a best case scenario, we were surprised to find that only about half remembered having discussions with their doctors about cancer screening. Furthermore, a minority believed that key pieces of information necessary for informed decision making, such as delayed benefits of cancer screening and the need to consider life expectancy to realize a benefit from screening, were important.

The participants' responses to the question, "What might make you stop cancer screening?" included age, deteriorating health, poor quality of life, or nearing death, concerns about the reliability of the tests, and doctors' recommendation. Resnick asked residents in several continuing care retirement communities the converse of this question, why they had not been screened. She found age, and lack of doctor recommendation as the most common reasons given [27,28]. She also evaluated predictors of screening test completion in these venues and found that increasing age and the number of chronic conditions were consistently predictors of not getting screening tests [27-29]. However, physician recommendation was not evaluated in these models and has been shown to be a significant influence on screening behavior [30-32].

Because physician recommendations are so influential, what role should they play in older adults where direct evidence supporting screening is lacking? Participants in this study were relatively healthy and most would likely benefit from screening. Their physicians may have recommended they have screening without further discussion. However, the importance of individualized decision making has been stressed for patients of all ages because few individuals who undergo screening actually benefit, while all are exposed to the potential risks of false positives and the burden of screening [3,33]. This burden may fall more heavily on older adults, making participation in decision making more important [34].

However, encouraging individualized decision making and discouraging physician recommendations against screening could result in screening among those least likely to benefit. Forty-three percent of our participants would consider screening even if their doctor recommended against it and the majority wanted to continue to screen throughout their lifetime. Although the right to refuse screening is well supported by ethical tenets, the ethics of continuing screening when the mortality benefit is unlikely is less certain [35]. A key limitation of our study is that it is not clear whether the enthusiasm for screening results from a lack of knowledge about the delay in mortality benefits from screening later in life, or because they value other benefits from screening such as a psychological benefit of alleviating fears of cancer, or would want aggressive treatment for cancers if found [36]. Additional research is needed to determine whether older adults adequately understand this information or use other determinants to make their decisions about cancer screening.

Caution should be used in generalizing our findings to other populations. Participants in our study were volunteers and may have chosen to participate because of a strong belief in cancer screening. Many also had a previous diagnosis of cancer. Consequently, our results may be biased in favor of cancer screening. However, we should point out that our findings are consistent with a nationally representative survey that found a similar enthusiasm for cancer screening [37]. Additionally, our participants were very well educated, reflecting the population of the continuing care retirement communities in our area. In the general population, only 15% of those age 70 and older had graduated from college in 1999 compared to 83% in our population [38]. Finally, although we cognitively tested the questionnaire, the questions we used have not been validated previously. Therefore, we cannot be certain that participants interpreted the questions as we intended.

## Conclusion

This study assessed older adults' perspectives on continuing cancer screening and individualized decision making for cancer screening. In this select group of older adults, many reported not having the opportunity to assess the risks, benefits, and uncertainties of cancer screening later in life. However, the majority of participants said that they would prefer to have discussions about the possibility of stopping screening with their physicians. Despite this preference, many participants were reluctant to discuss life expectancy in this context and did not believe that it was important. Participants held positive attitudes about continuing cancer screening later in life, and many may have had unrealistic expectations about the potential survival benefits of screening. Future research is needed to determine whether educating older adults about the importance of longevity in screening decisions would be acceptable, affect older adults' attitudes about screening, or change their screening behavior.

## Declaration of competing interests

The author(s) declare that they have no competing interests.

## Authors' contributions

CL conceived of the study, analyzed the data, and drafted the manuscript. CK and HA helped to design the survey, acquired the data, and helped in the analysis and interpretation. DB contributed to data acquisition, study conception, survey design, and data interpretation. LW and MP contributed to the conception of the study, survey design, and interpreting the data. All authors helped to draft the manuscript, revised it critically and gave final approval of the manuscript.

## Pre-publication history

The pre-publication history for this paper can be accessed here:


